# Serum vitamin D and B9 are positively associated with muscle mass in young and middle-aged adults: a cross-sectional study

**DOI:** 10.3389/fnut.2026.1866326

**Published:** 2026-07-14

**Authors:** Xi-Peng Liu, Juan Sun, Zhe Liu, Hai-Feng Zhang, Jie-Wen Chen

**Affiliations:** Department of Clinical Nutrition, Shanghai Ninth People's Hospital, Shanghai JiaoTong University School of Medicine, Shanghai, China

**Keywords:** BIA-derived ASM/BMI, cross-sectional study, FNIH criteria, serum vitamin levels, young and middle-aged adults

## Abstract

**Objectives:**

This cross-sectional study aimed to investigate associations between serum levels of multiple vitamins (D, E, B1, B3, B6, B9) and muscle mass measured as BIA-derived appendicular skeletal muscle mass adjusted by body mass index (ASM/BMI) in young and middle-aged Chinese adults.

**Methods:**

A total of 534 participants aged 18–55 years were recruited. Serum vitamins were measured using liquid chromatography-tandem mass spectrometry (LC-MS/MS). ASM/BMI was derived from bioelectrical impedance analysis (BIA). Multivariate linear and ordinal logistic regression models were used adjusted for age, gender, lifestyle factors, nutritional supplementation, and chronic diseases. False discovery rate (FDR) correction was applied for multiple testing. Subgroup analyses were conducted by gender and age (18–30 vs. 30–55 years).

**Results:**

In adjusted linear regression, serum vitamin D [*B* = 0.003, 95% CI (0.001–0.004), *p* < 0.001] and vitamin B9 [*B*=0.002, 95% CI (0.000–0.003), *p* = 0.015] were positively associated with ASM/BMI. Ordinal logistic regression confirmed that serum vitamin D [OR = 1.044, 95%CI (1.018, 1.070), *p*=0.001] and B9 [OR = 1.031, 95%CI (1.005, 1.059), *p* = 0.020] were associated with higher odds of being in the higher ASM/BMI quartile. Vitamin B1 showed a negative association in linear regression [B = −0.007, 95% CI (−0.012, −0.002), FDR-*p* = 0.012] but did not survive FDR correction in logistic models (FDR-*p* = 0.084). Sensitivity analyses using ASM/height^2^ yielded opposite results vitamin B9 became negatively associated with muscle mass (B=-0.019, *p*=0.003), and the positive associations for vitamin D were no longer observed, highlighting the importance of normalization method.

**Conclusions:**

In this cross-sectional study, higher serum vitamin D and vitamin B9 were associated with BIA-derived ASM/BMI. The negative association for vitamin B1 was not robust after FDR correction. These hypothesis-generating findings require prospective validation. Clinical trial registration number: ChiCTR2600124808 (China Clinical Trial Registry).

## 1 Introduction

Low muscle mass is one of the main characteristics of sarcopenia, which is a syndrome characterized by the decline in muscle mass and function associated with aging. It is typically seen in the elderly and can lead to physical dysfunction, decreased quality of life, and an increased risk of death (1–3). Recent studies have shown that the incidence of low muscle mass among young and middle-aged people is also gradually increasing. This may be closely related to unhealthy lifestyles of young and middle-aged people, such as sedentariness, lack of exercise, high work stress, and unbalanced dietary nutrition (4). Insufficient intake or absorption of protein, energy and trace elements is closely related to the reduction of muscle mass (5). Therefore, nutritional intervention is one of the important intervention measures for preventing and treating sarcopenia. Currently, in addition to the focus on the prevention and treatment effects of protein-energy metabolism and essential amino acids and their metabolites on muscle loss, more and more studies are also paying attention to the relationship between vitamins and muscle loss (6, 7).

Vitamins play a significant role in regulating muscle mass by promoting protein synthesis, inhibiting the breakdown of muscle fibers, participating in glycogen metabolism to provide energy support for muscle growth, and reducing oxidative damage to muscle cells (8–11). Although some studies have found that there might be a certain correlation between vitamins and muscle mass, studies on the relationship between vitamin blood concentrations and muscle mass are limited and inconsistent, and previous studies have mostly focused on vitamin D. A cross-sectional study of the Chinese population showed that low serum vitamin D levels were associated with reduced muscle mass in men (12). Two systematic reviews indicated that supplementing with vitamin D might have a positive effect on muscle function in elderly individuals with low baseline serum vitamin D levels (13, 14). However, another systematic review reached the opposite conclusion (15). Evidence on other vitamins is even scarcer. A cross-sectional study reported lower vitamin B6 and B9 intake in sarcopenic elderly (16), and a UK study linked vitamin E intake to lean mass percentage in women (17). Nevertheless, these studies are limited by single-variable designs, elderly populations, and reliance on dietary intake rather than serum biomarkers.

Currently, the conventional method for assessing the health status of muscle mass is the traditional skeletal muscle index (SMI = ASM/height^2^), which is recommended by AWGS 2019 and EWGSOP2 (1, 18). However, it is highly correlated with BMI, resulting in a systematic underestimation of muscle mass in overweight/obese populations and an overdiagnosis in lean individuals. To address this, the FNIH Sarcopenia Project (19) introduced ASM/BMI as an alternative that better accounts for overall body size. The AWGS 2025 consensus (20) formally incorporated ASM/BMI into its diagnostic algorithm for Asian populations, noting that it “better reflects the influence of body habitus on muscle mass.” In this cross-sectional study, the study population was young to middle-aged individuals with a high proportion of overweight/obese individuals. Therefore, we used ASM/BMI as our primary outcome and repeated analyses with ASM/height^2^ for sensitivity.

Knowledge gap and innovation of this study: To date, no study has systematically examined the associations between a broad panel of serum vitamins (D, E, B1, B3, B6, B9) and ASM/BMI in young and middle-aged adults—a critical period for achieving peak muscle mass and initiating early prevention. This cross-sectional study provides hypothesis-generating evidence on these associations.

## 2 Materials and methods

### 2.1 Participants

**Figure 1:**
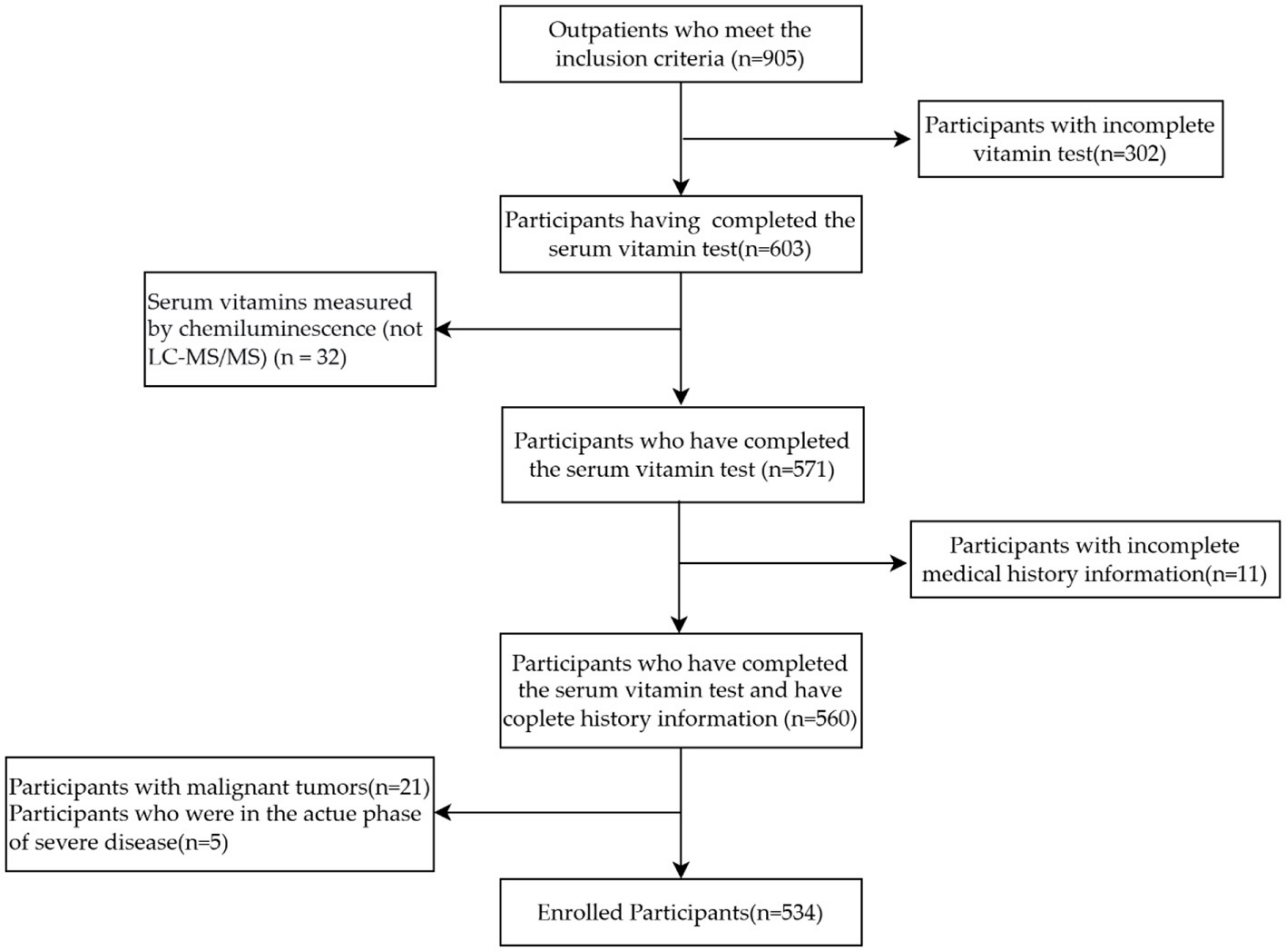
The flow chart for the cross-sectional study.

A total of 534 participants were recruited from clinical nutrition department outpatient clinic of the Ninth People's Hospital Affiliated to Shanghai Jiao Tong University School of Medicine in Shanghai, China, from April 2024 to September 2025 (Figure 1). The inclusion criteria were as follows: (i) Chinese; (ii) 18 years ≤ age ≤ 55 years; (iii) Body composition analysis measurement (BIA) has been conducted; (iv) No participation in any other clinical studies within the past 3 months. Exclusion criteria were as follows: (i) Patients with malignant tumors or were in the acute phase of severe diseases (e.g., cancer, cardiovascular and cerebrovascular diseases, and/or psychiatric diseases); (ii) Serum vitamin level test has not been completed; (iii) Serum vitamin levels were not quantified using the standardized liquid chromatography-tandem mass spectrometry (LC-MS/MS) method; and (iv) The medical history information is incomplete (including smoking and drinking habits, leisure-time physical activity, use of dietary supplements, and chronic disease history, etc.). The study adhered to the ethical guidelines of the 1975 Declaration of Helsinki and was approved by the institutional review board of the Ninth People's Hospital Affiliated to Shanghai Jiao Tong University School of Medicine (SH9H-2026-T212-1). The institutional ethics committee approved a waiver of informed consent for this retrospective cross-sectional study, as the research posed no more than minimal risk to participants and the waiver did not adversely affect their rights or welfare, given the important scientific value of the study. Research registration information: This research has been registered in the National Medical Research Registration and Filing Information System (Registration Number: MR-31-26-033323).

### 2.2 Assessment of vitamin levels

Patients' Blood samples were collected after an overnight fast (at least 12 h). All blood samples were tested in medical laboratory center of the Ninth People's Hospital Affiliated to Shanghai Jiao Tong University School of Medicine. The serum samples were used to test the concentration of serum vitamins. The principal study variables included concentrations of vitamins D, E, B1, B3, B6 and B9. Liquid chromatography with tandem mass spectrometry (LC-MS/MS) was used to quantified all of the above vitamins.

### 2.3 Body composition and assessment of muscle mass

All participants underwent standard physical measurements. Height was obtained with participants wearing light clothing and no shoes. Bioelectrical impedance analysis (BIA, MC-180, TANITA, Japan) was used to assess body composition. Appendiceal skeletal muscle mass (ASM) was calculated as the sum of lean masses of arms and legs. BMI was calculated as weight (kg)/height/(m^2^). The primary muscle mass index was ASM/BMI, following the FNIH Sarcopenia Project (19) and AWGS 2025 (20) recommendations. This index reduces confounding by overall body size and adiposity.

For sensitivity, we repeated all analyses using the conventional ASM/height^2^ (1, 18).

### 2.4 Covariates

While assessing the relationship between serum vitamins and ASM/BMI in young and middle-aged participants, risk factors related to ASM/BMI were selected and controlled. The following covariates were collected and considered: age (continuous variable), gender (female or male), smoking status (current smoker or non-smoker), alcohol status (current drinker or non-drinker), and leisure-time physical activity (inactive, moderate/vigorous), nutritious supplementary (current supplement takers, non-supplement takers) and chronic diseases (yes/no). The date of birth, gender, smoking status, drinking status, leisure-time physical activity, nutritious supplementary and clinical history of the young and middle-aged patients were collected by reviewing the medical records. The disease information included hyperlipidemia (self-report or physician diagnoses), diabetes (self-reported and/or fasting blood glucose 7.0 mmol/L and/or random blood glucose 11.1 mmol/L), hypertension (self-reported and/or systolic blood pressure 140 mmHg and/or diastolic blood pressure 90 mmHg) and cardiovascular disease (self-reported or physician diagnoses). These diseases are regarded as potential interfering factors. If an individual has one or more than one disease, they are defined as having a chronic condition. The selection of covariates is based on previously published studies and the available variables (21, 22).

### 2.5 Statistical analysis

Continuous variables were tested for normality using the Kolmogorov-Smirnov test. Since the data were skewed, all measurement data were expressed using the median and interquartile range M (P25, P75), and the comparison between groups is conducted using a non-parametric test (Wilcoxon test). Categorical variables were presented as the numbers (percentages), and comparisons between groups are conducted using the Chi-square test.

Multivariate linear regression was used to estimate B coefficients (*B*) and 95% confidence intervals (CIs) for the association between each serum vitamin and ASM/BMI. Two models were constructed: Model 1 (unadjusted) and Model 2 (adjusted for age, gender, smoking status, drinking status, leisure-time physical activity, nutritious supplementary, and chronic diseases). To examine the change in the coefficient of vitamin B9 from crude to adjusted models, we performed stepwise adjustment by sequentially adding covariates in the following order: Model 1 (unadjusted), Model 2 (+age), Model 3 (+age +gender), Model 4 (+ age+ gender+ lifestyle factors: smoking, drinking, physical activity), Model 5 (+ age+ gender+ lifestyle factors +nutritional supplement), Model 6 (+ age+ gender+ lifestyle factors +nutritional supplement +chronic diseases). The change in the B9 coefficient and its significance were tracked across these models.

To formally test whether the association between vitamin B9 and ASM/BMI differs by gender, we added a product term (vitamin B9 × gender) to the fully adjusted linear regression model (Model 2). The interaction was considered significant at *p* < 0.05.

To assess associations across the full distribution of ASM/BMI, we used ordinal logistic regression with the four quartiles (Q1–Q4) as the ordered outcome. The proportional odds assumption was verified (test of parallel lines, *p* > 0.05). Odds ratios (ORs) represent the change in odds of being in a higher quartile per unit increase in the independent variable. Multicollinearity was assessed using variance inflation factors (VIF) from a linear model containing all predictors; VIF > 5 considered problematic. Pearson correlations among vitamins were also computed. Because we tested six vitamins in two regression models (12 primary tests), we applied Benjamini-Hochberg false discovery rate (FDR) correction (α = 0.05) using R software (version 4.3.1; R Foundation for Statistical Computing, Vienna, Austria) with the *p*-adjust function and method = “BH.” Adjusted p-values (FDR-p) are reported in Supplementary Table S3.

Subgroup analyses were stratified by sex and age (18–30 vs. 30–55 years). For strata with sparse data (e.g., < 10 participants in a covariate category), that covariate was excluded to avoid model instability.

All primary regression analyses (linear and ordinal logistic) were performed using SPSS 25.0. Forest plots were drawn using GraphPad Prism. Full model results (including non-significant variables) are presented in Supplementary Figures S1, S2.

There were no missing data for the primary exposure variables (serum vitamins), outcome (ASM/BMI), or covariates included in the regression models. Therefore, a complete-case analysis was performed using all 534 participants. No imputation methods were required.

## 3 Results

### 3.1 Participants' characteristics

This study included 534 participants (76.4% were female). The average age of these participants was 32.6 ± 7.4 years (median 32 year, ranged 18–55 years). Table 1 presents baseline characteristics stratified by ASM/BMI. Participants in the highest ASM/BMI quartile were more likely to be male (78.6%), younger, have lower chronic disease prevalence, higher serum vitamin D and B6 levels, and engage in regular physical activity (all *p* < 0.05). Furthermore, in the group with higher levels of ASM/BMI, the proportions of smokers and drinkers are also higher (*p* = 0.000). This might be due to the fact that the higher ASM/BMI group has a higher proportion of males. No significant differences were observed across quartiles in nutritional supplement use or serum levels of vitamins E, B1, B3, and B9 in the crude comparison (*p* > 0.05).

**Table 1:** Participant characteristics by level of ASM/BMI.

M (P25,P75)/n (%)	Total (*N*=534)	Level of ASM/BMI				*p* value
		Quartile 1	Quartile 2	Quartile 3	Quartile 4	
ASM/BMI (range)		0.51~0.68	0.69~0.76	0.77~0.88	0.89~1.36	
Age (y)	32 (27, 37)	35 (29, 40)	34 (29, 39)	30 (26, 35)	30 (26, 35)	0.000
Gender						0.000
Male	126 (23.6%)	0 (0%)	4 (3.1%)	19 (14.8%)	103 (78.6%)	
Female	408 (76.4%)	144 (100%)	127 (96.9%)	109 (85.2%)	28 (21.4%)	
Smoking status						0.000
Smoker	21 (3.9%)	2 (1.4%)	1 (0.8%)	4 (3.1%)	14 (10.7%)	
Non-smoker	513 (96.1%)	142 (98.6%)	130 (99.2)	124 (96.9%)	117 (89.3%)	
Drinking status						0.000
Drinker	30 (5.6%)	3 (2.1%)	5 (3.8%)	3 (2.3%)	19 (14.5%)	
Non-drinker	504 (94.4%)	141 (97.9%)	126 (96.2%)	125 (97.7%)	112 (85.5%)	
Leisure-time physical activity						0.001
Inactive	396 (74.2%)	119 (82.6%)	92 (70.2%)	102 (79.7%)	83 (63.4%)	
Active	138 (25.2%)	25 (17.4%)	39 (29.8%)	26 (20.3%)	48 (36.6%)	
Nutritious supplementary						0.937
Current supplement takers	43 (8.1%)	11 (7.6%)	12 (9.2%)	9 (7.0%)	11 (8.4%)	
Non-supplement takers	491 (91.9%)	133 (92.4%)	119 (90.8%)	119 (93.0%)	120 (91.6%)	
Chronic disease						0.017
Yes	138 (25.8%)	46 (31.9%)	35 (26.7%)	20 (15.6%)	37 (28.2%)	
No	396 (74.2%)	98 (68.1%)	96 (73.3%)	108 (84.4%)	94 (71.8%)	
Vitamin D (ng/ml)	17.5 (13.2, 22.5)	16.1 (12.2, 21.1)	17.5 (13.3, 22.7)	16.8 (13.2, 21.8)	20.2 (14.5, 24.1)	0.003
Vitamin E (ug/ml)	8.86 (7.0, 11.2)	8.9 (7.1, 10.9)	8.3 (6.8, 11.5)	9.2 (7.2, 11.4)	9.0 (7.1, 11.3)	0.493
Vitamin B1 (ng/ml)	3.1 (2.2, 4.6)	3.4 (2.4, 4.9)	3.1 (2.2, 4.9)	3.0 (2.2, 4.4)	2.8 (2.2, 4.5)	0.091
Vitamin B3 (ng/ml)	21.6 (15.6, 30.1)	23.5 (16.6, 31.3)	22.3 (16.8, 27.7)	19.4 (14.0, 29.8)	21.9 (15.3, 31.9)	0.155
Vitamin B6 (ng/ml)	3.3 (2.9, 4.5)	3.1 (2.4, 3.9)	3.2 (2.6, 4.2)	3.3 (3.0, 4.8)	3.9 (3.1, 5.4)	0.000
Vitamin B9 (ng/ml)	8.8 (6.2, 13.5)	9.7 (6.1, 13.5)	9.0 (6.4, 12.1)	9.0 (6.8, 16.4)	7.7 (5.8, 12.4)	0.069

### 3.2 Multivariate liner regression

We constructed two multivariable linear regression models (Table 2). In model 2 (fully adjusted), serum vitamin D (*B* = 0.003, 95%CI: 0.001–0.004, *p* = 0.000) and vitamin B9 (*B* = 0.002, 95%CI: 0.000–0.003, *p* = 0.015) were positively associated with ASM/BMI. Vitamin B1 showed a negative association (*B* = −0.007,95%CI: −0.012 to −0.002, *p* = 0.003) and ASM/BMI. No significant associations were found for vitamins E, B3, or B6.

**Table 2:** Association between serum vitamins and ASM/BMI (multivariate linear regression).

Variates	Models	*B* (95% CI)	*B* standard error	*t*	*p* value
Vitamin D (ng/ml)	Model 1	0.004 (0.002, 0.006)	0.001	4.459	0.000
	Model 2	0.003 (0.001, 0.004)	0.001	4.195	0.000
Vitamin E (ug/ml)	Model 1	0.001 (−0.004, 0.006)	0.002	0.464	0.642
	Model 2	0.000 (−0.003, 0.003)	0.002	−0.075	0.941
Vitamin B1 (ng/ml)	Model 1	−0.008 (−0.016, −0.001)	0.004	−2.196	0.029
	Model 2	−0.007 (−0.012, −0.002)	0.002	−2.962	0.003
Vitamin B3 (ng/ml)	Model 1	0.001 (0.000, 0.002)	0.001	1.173	0.241
	Model 2	0.000 (−0.001, 0.001)	0.000	−0.506	0.613
Vitamin B6 (ng/ml)	Model 1	0.001 (−0.001, 0.003)	0.001	0.735	0.463
	Model 2	−5.830E-6 (−0.002.0.001)	0.001	−0.008	0.994
Vitamin B9 (ng/ml)	Model 1	−0.002 (−0.004, 0.000)	0.001	−1.625	0.105
	Model 2	0.002 (0.000, 0.003)	0.001	2.440	0.015

Original *p*-values are shown (*n* = 534). For FDR-adjusted *p*-values and significance thresholds, refer to Supplementary Table S3.

Model 1: unadjusted. Model 2: adjusted for age, sex, smoking status, drinking status, leisure-time physical activity, nutritional supplement, and chronic diseases.

After FDR correction (Supplementary Table S3), vitamin D and B9 remained significant in both linear and ordinal models; vitamin B1 was significant only in the linear model (FDR-*p* = 0.012) but not in the ordinal logistic model (FDR-*p* = 0.084).

Collinearity diagnostics (Supplementary Tables S1, S2): All inter-vitamin Pearson correlations were < 0.34, and all VIF values < 2.1, indicating no problematic multicollinearity.

### 3.3 Ordinal logistic regression

Ordinal logistic regression (Table 3) showed that higher serum vitamin D (OR = 1.044, 95% CI: 1.018–1.070, *p* = 0.001) and vitamin B9 (OR = 1.031, 95% CI: 1.005–1.059, *p* = 0.020) were associated with higher odds of being in the higher ASM/BMI quartile. Vitamin B1 was associated with lower odds (OR = 0.907, 95% CI: 0.825–0.997, *p* = 0.042) but after FDR correction this association did not remain significant (FDR-*p* = 0.084).

**Table 3:** Association between serum vitamins and ASM/BMI quartile categories (ordinal logistic regression).

Variates	*OR* (95% *CI*)	*p* value
Vitamin D	1.044 (1.018, 1.070)	0.001
Vitamin E	1.030 (0.968, 1.096)	0.348
Vitamin B1	0.907 (0.825, 0.997)	0.042
Vitamin B3	0.988 (0.974, 1.002)	0.083
Vitamin B6	1.000 (0.971, 1.030)	0.986
Vitamin B9	1.031 (1.005, 1.059)	0.020
Age	0.972 (0.948, 0.997)	0.026
Female	0.011 (0.006, 0.021)	0.000
Male		
Non-smoker	1.055 (0.276, 4.026)	0.938
Smoker		
Non-drinker	1.041 (0.340, 3.185)	0.944
Drinker		
Non-supplement takers	1.886 (0.950, 3.744)	0.070
Supplement takers		
Leisure-time physical activity		
Inactive	0.896 (0.599, 1.342)	0.595
Active		
Chronic disease		
No	2.316 (1.524, 3.521)	0.000
Yes		

Original *p*-values are presented (n=534). For FDR-adjusted *p*-values and significance thresholds, refer to Supplementary Table S3.

The proportional odds assumption was satisfied (test of parallel lines, χ^2^ = 34.31, df = 26, *p* = 0.127). Nagelkerke pseudo *R*^2^ was 0.518.

### 3.4 Sensitivity analysis using ASM/height^2^

When ASM/height^2^ was used as the outcome (Supplementary Tables S4, S5), the associations reversed. In multivariate linear regression, vitamin D was not significantly associated (B = −0.006, 95%CI: −0.017–0.006, *p* = −0.037), vitamin B9 was negatively associated (B = −0.019, 95%CI: −0.031 to −0.006, *p* =-0.003), and vitamin B1 showed a positive but non-significant trend (B = 0.017, 95%CI: −0.026–0.061, *p* =-0.432). Ordinal logistic regression confirmed that higher vitamin B9 was associated with lower odds of being in a higher SMI quartile (OR = 0.968, 95% CI: 0.825–0.997, *p* = 0.012). These opposite results underscore that the choice of normalization method critically influences findings, and support the use of ASM/BMI in populations with heterogeneous body size.

### 3.5 Subgroup analyses by gender and age

**Figure 2:**
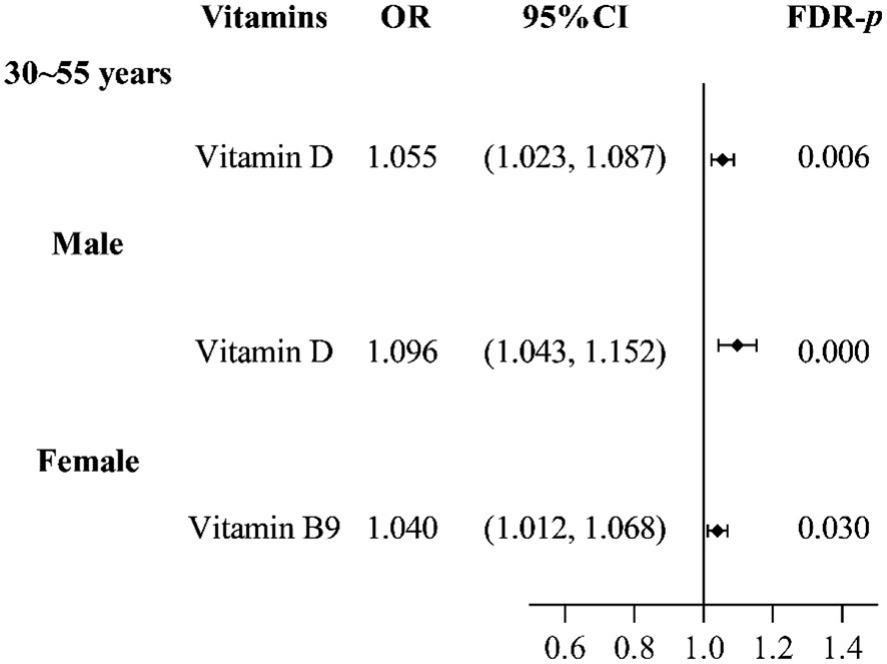
Forest plot of significant associations between serum vitamins and ASM/BMI quartile categories in subgroup analyses after false discovery rate (FDR) correction. Only vitamins with FDR-adjusted *p* < 0.05 within each subgroup are displayed. For the younger subgroup (< 30 years), no vitamin met the significance threshold.

The stratified regression analyses (Figure 2) were conducted for different gender and age groups. In the younger age group (< 30 years), only seven participants reported smoking and nine participants reported drinking. Therefore, smoking and drinking status was not included as a covariate in the ordinal logistic regression for this subgroup. The results for other covariates remained unchanged.

Gender subgroups (Supplementary Figure S1): Among females (*n* = 408), higher serum vitamin B9 was significantly associated with higher odds of being in a higher ASM/BMI quartile (OR = 1.040, 95%CI: 1.012–1.068, *p* = 0.005; FDR-*p* = 0.030). Among males (*n* = 126), serum vitamin D showed a positive association (OR = 1.096, 95%CI: 1.043–1.152, *p* < 0.001; FDR-*p* < 0.001). No other vitamins reached significance after FDR correction (Supplementary Table S6).

Age subgroups (Supplementary Figure S2): In the middle-aged group (30–55 years, *n* = 341), vitamin D (OR = 1.055, 95%CI: 1.023–1.087, *p* = 0.001; FDR-*p* = 0.006) and vitamin B9 (OR = 1.035, 95%CI: 1.005–1.066, *p* = 0.021; FDR-*p* = 0.063) showed positive associations; vitamin B9 did not survive FDR correction. In the younger group (< 30 years, *n* = 184), no vitamin remained significant after FDR correction (Supplementary Table S6).

**Explanation for vitamin B9 coefficient change (****Supplementary Table S7**, **S8****):** Vitamin B9 was not significant in unadjusted linear regression (*B* = −0.002, *p* = 0.105). After adding age and gender, the coefficient became positive and significant. This change was driven primarily by gender: females had lower ASM/BMI but similar or higher vitamin B9 levels compared to males (Table 1). Formal interaction test between vitamin B9 and gender was not significant (*p* = 0.710, Supplementary Table S8), indicating that the effect is due to strong main effects of gender rather than true interaction.

## 4 Discussion

Muscle mass increases rapidly during the period from 3 to 18 years as the human body grows and develops. It reaches its peak around the age of 30, and then muscle strength and mass decline at a rate of 0.5%−1% per year (23). However, previous studies have mostly focused on the elderly population and there is a scarcity of research on the association between serum vitamin levels and muscle mass in young and middle-aged groups. This cross-sectional study investigated associations between six serum vitamins and BIA-derived ASM/BMI in young and middle-aged Chinese adults. The main findings are: (i) higher vitamin D and B9 levels are positively associated with ASM/BMI, with associations robust to multiple testing correction; (ii) the negative association observed for vitamin B1 did not survive FDR correction in logistic models and should be considered exploratory; (iii) the choice of muscle mass normalization method (ASM/BMI vs. ASM/height^2^) dramatically influences results, with ASM/height^2^ yielding opposite or null findings.

### 4.1 Vitamin D and muscle mass

The positive association between vitamin D and ASM/BMI was consistent across models and remained significant after FDR correction. This result is highly consistent with a recent Mendelian randomization study involving 410,000 people which was the first to confirm a causal relationship between serum 25(OH)D levels and limb muscle mass, and it also found that the effect was more significant in men (24). Mechanistically, vitamin D activates vitamin D receptors in muscle cells, regulating genes involved in protein synthesis and inhibiting ubiquitin-proteasome-mediated breakdown (25, 26). However, our observational findings cannot establish causality and large RCTs such as DO-HEALTH (27) found no effect of vitamin D supplementation alone on muscle mass in healthy elderly. Thus, our results are hypothesis-generating and do not imply that supplementation will increase muscle mass.

**Clinical relevance of effect size:** The linear coefficient *B* = 0.003 for vitamin D implies that a 10 ng/ml increase in serum vitamin D is associated with a 0.03 increase in ASM/BMI. Given that ASM/BMI in our sample ranged from 0.51 to 1.36, this effect size is small and may not be clinically significant at the individual level. However, even small differences at the population level could be relevant for risk stratification.

### 4.2 Vitamin B9 (folate) and muscle mass

Vitamin B9 showed a positive association with ASM/BMI only after adjustment for age and sex, with the change driven by sex (females had lower muscle mass but similar B9 levels). This is consistent with previous studies linking dietary folate to muscle strength (16, 28). Folate participates in one-carbon metabolism and DNA methylation, potentially influencing muscle-related gene expression. The lack of a significant interaction between B9 and gender (*p* = 0.710) suggests that the effect modification is due to the strong main effect of sex rather than a true biological interaction.

### 4.3 Vitamin B1: an exploratory negative finding

In contrast to previous studies reporting a protective association between vitamin B1 and muscle mass in elderly, diabetic, or deficient populations (29–31), our study found no robust independent relationship between serum vitamin B1 and ASM/BMI in healthy young and middle-aged adults. In our cross-sectional study, serum vitamin B1 was negatively associated with ASM/BMI in linear regression (*p* = 0.003) but not in ordinal logistic regression after FDR correction (FDR-*p* = 0.084). Additionally, the crude quartile comparison in Table 1 showed no significant difference (*p* = 0.091).

Several factors may explain this discrepancy. First, residual confounding cannot be excluded—we did not collect data on dietary patterns, total energy and carbohydrate intake, alcohol consumption, renal function, physical activity, or the type/dose of vitamin supplements, all of which could influence both circulating B1 levels and muscle mass. Second, serum vitamin B1 may not accurately reflect tissue-level utilization or long-term nutritional status, whereas previous positive studies relied primarily on dietary intake, a measure of habitual exposure. Third, and most importantly, the protective effects of vitamin B1 on muscle mass have predominantly been documented in deficiency-prone populations, particularly the elderly, who often experience reduced appetite, malabsorption, and medication interference (e.g., diuretics), leading to marginal B1 deficiency. In such contexts, supplementation restores energy metabolism and IGF-I signaling, yielding a clear muscle-protective effect. In contrast, our relatively young and well-nourished participants likely have adequate vitamin B1 status, placing them on the plateau of the dose-response curve where further variations in serum B1 no longer translate into differences in relative muscle mass. Therefore, while vitamin B1 is essential for muscle homeostasis under deficient conditions, it does not appear to be an independent determinant of muscle mass in healthy, adequately nourished young-to-middle-aged adults.

### 4.4 Methodological implications: ASM/BMI vs. ASM/height^2^

A key contribution of this study is the direct comparison of two normalization methods. When we used ASM/height^2^ (the conventional SMI), the positive associations for vitamin D and B9 disappeared, and vitamin B9 became negatively associated (Supplementary Tables S4, S5). This reversal is consistent with the known deviation of ASM/height^2^. This method is highly correlated with BMI, resulting in systematic underestimation of muscle mass in overweight/obese individuals, while overestimation occurs in slender individuals (17, 19). In our cohort, the highest ASM/BMI quartile contained more males and younger participants-characteristics associated with higher BMI. ASM/BMI effectively removed this body-size bias, revealing associations that align with biological plausibility. These findings support the FNIH (19) and AWGS 2025 (20) recommendations to incorporate BMI adjustment when evaluating muscle mass in populations with wide BMI ranges. We therefore encourage future studies to report both indexes or, at minimum, justify their choice based on the population's adiposity profile.

### 4.5 Strengths and limitations

**Strengths:** (i) for the first time, a systematic assessment was conducted in Chinese young and middle-aged adults to investigate the association between multiple serum vitamins and muscle quality indicators; (ii) the vitamin levels were precisely determined using LC-MS/M; (iii) comprehensive covariate adjustment; (iv) FDR correction; (v) ordinal logistic regression; (vi) sensitivity analysis with ASM/height^2^.

**Limitations:** (i) Cross-sectional design precludes causal inference; all findings are associational; (ii) Residual confounding is likely: we did not measure dietary protein or energy intake, sunlight exposure, alcohol quantity, renal function, or specific supplement dosages. These factors could influence both vitamin status and muscle mass; (iii) Single-center clinic sample with 76.4% female limits generalizability to men and the general population. Subgroup analyses for males (*n* = 126) are underpowered; (iv) BIA is less accurate than DXA, though acceptable for epidemiological studies; (v) Multiple testing was addressed with FDR, but some subgroup findings may still be false positives; (vi) The small effect sizes (e.g., *B* = 0.003 for vitamin D) suggest that even if associations are real, the clinical relevance is modest.

## 5 Conclusions

In this cross-sectional study of young and middle-aged Chinese adults, higher serum vitamin D and B9 levels are positively associated with BIA-derived ASM/BMI. The negative association observed for vitamin B1 was not robust after multiple testing correction. The choice of muscle mass normalization method substantially influences findings; ASM/BMI appears preferable to ASM/height^2^ in populations with heterogeneous body size. These results are hypothesis-generating and require confirmation in prospective cohort studies before any causal or interventional inferences can be made.

## Data Availability

The raw data supporting the conclusions of this article will be made available by the authors, without undue reservation.
