# Supplementary material for: Serum vitamin D and B9 are positively associated with muscle mass in young and middle-aged adults: a cross-sectional study
Source: Front Nutr. 2026 Jul 14;13:1866326. doi: 10.3389/fnut.2026.1866326 (PMC13407269; doi:10.3389/fnut.2026.1866326)
Supplement: Supplementary file 1 [file Data_Sheet_1.pdf]

Supplementary Online Content

Supplement Table S1. Pearson correlation matrix among the six serum vitamins

Supplement Table S2. Multicollinearity diagnostics in the fully adjusted linear regression model (Model 2)

Supplement Table S3. Benjamini-Hochberg false discovery rate (FDR) correction to the 12 p-values from the primary linear and ordinal logistic regression models (six vitamins each)

Supplementary Table S4. Multivariable linear regression analysis of SMI (ASM/height^2) with serum vitamins and covariates

Supplementary Table S5. Ordinal logistic regression analysis of SMI quartile categories with serum vitamins and covariates

Supplement Table S6. FDR correction for subgroup analyses (ordinal logistic regression)

Supplement Table S7. Stepwise changes in the coefficient of vitamin B9 in linear regression models predicting ASM/BMI

Supplementary Table S8. Interaction between vitamin B9 and sex in the fully adjusted linear regression model

Supplementary Figure S1 Complete forest plots of gender-stratified ordinal logistic regression (all variables).

Supplementary Figure S2 Complete forest plots of age-stratified ordinal logistic regression (all variables).

Supplement Table S1. Pearson correlation matrix among the six serum vitamins

Vitamins		Vitamin D	Vitamin E	Vitamin B1	Vitamin B3	Vitamin B6	Vitamin B9
Vitamin D	Pearson correlation	1	0.161	0.145	-0.017	0.260	0.230
	p value		0.000	0.001	0.691	0.000	0.000
Vitamin E	Pearson correlation	0.161	1	0.096	0.018	0.093	0.211
	p value	0.000		0.026	0.677	0.032	0.000
Vitamin B1	Pearson correlation	0.145	0.096	1	0.279	0.334	0.204
	p value	0.001	0.026		0.000	0.000	0.000
Vitamin B3	Pearson correlation	-0.017	0.018	0.279	1	0.021	-0.018
	p value	0.691	0.677	0.000		0.635	0.673
Vitamin B6	Pearson correlation	0.260	0.093	0.334	0.021	1	0.265
	p value	0.000	0.032	0.000	0.635		0.000
Vitamin B9	Pearson correlation	0.230	0.211	0.204	-0.018	0.265	1
	p value	0.000	0.000	0.000	0.673	0.000	

Note: All correlations are weak to moderate; the highest is between B1 and B6 ($r = 0.3334$), well below the collinearity threshold ($r > 0.70$).

Supplement Table S2. Collinearity diagnostics (VIF and Tolerance) for fully adjusted linear regression model (Model 2)

	Collinearity statistics	
Variate	Tolerance values	VIF
Age	0.812	1.232
Sex	0.819	1.221
Smoking status	0.491	2.038
Drinking status	0.488	2.048
Leisure-time physical activity	0.933	1.072

Nutritious supplementary	0.828	1.208
Chronic diseases	0.874	1.145
Vitamin D	0.780	1.282
Vitamin E	0.822	1.216
Vitamin B1	0.789	1.267
Vitamin B3	0.888	1.126
Vitamin B6	0.785	1.274
Vitamin B9	0.819	1.221

Note: All VIF values are below 2.1, well under the commonly used threshold of 5 or 10. Even the highest VIF (2.048 for drinking status) indicates only mild collinearity, which does not bias coefficient estimates or inflate standard errors to a problematic degree.

Supplement Table S3. Benjamini-Hochberg false discovery rate (FDR) correction to the 12 p-values from the primary linear and ordinal logistic regression models (six vitamins each)

Vitamins	Model	Original *p*	FDR-adjusted *p*	Significant after FDR (α=0.05)
Vitamin D	Linear	0.000	0.000	Yes
	Ordinal logistic	0.001	0.006	Yes
Vitamin E	Linear	0.941	0.994	No
	Ordinal logistic	0.348	0.522	No
Vitamin B1	Linear	**0.003**	**0.012**	**Yes***
	Ordinal logistic	**0.042**	**0.084**	**No**
Vitamin B3	Linear	0.613	0.817	No
	Ordinal logistic	0.083	0.142	No
Vitamin B6	Linear	0.994	0.994	No
	Ordinal logistic	0.986	0.994	No
Vitamin B9	Linear	0.015	0.045	Yes
	Ordinal logistic	0.020	0.048	Yes

* Note: Significant only in the linear model, not significant in the ordinal logistic model.

Supplementary Table S4. Multivariable linear regression analysis of SMI (ASM/height^2) with serum vitamins and covariates

Variates	B(95%CI)	Standard Error	t	*p*-value
Vitamin D	**-0.006(-0.017, 0.006)**	0.006	-0.962	**-0.337**

Vitamin E	-0.013(-0.041, 0.016)	0.015	-0.881	0.379
Vitamin B1	**0.017(-0.026, 0.061)**	0.022	0.787	**0.432**
Vitamin B3	0.003(-0.003, 0.010)	0.003	1.001	0.317
Vitamin B6	0.000(-0.013, 0.014)	0.007	0.028	0.978
Vitamin B9	**-0.019(-0.031, -0.006)**	0.006	-3.021	**0.003**
Age	-0.023(-0.035, -0.011)	0.006	-3.885	0.000
Sex	2.263(2.060, 2.467)	0.104	21.859	0.000
Smoking status	-0.127(-0.701, 0.448)	0.292	-0.433	0.665
Drinking status	0.141(-0.344, 0.627)	0.247	0.572	0.568
Leisure-time physical activity	0.253(0.068, 0.437)	0.094	2.684	0.008
Nutritious supplementary	0.082(-0.234, 0.397)	0.161	0.508	0.612
Chronic diseases	0.330(0.139, 0.521)	0.097	3.395	0.001

Note: SMI, skeletal muscle index (ASM/height^2).

Supplementary Table S5. Ordinal logistic regression analysis of SMI (ASM/height^2) quartile categories with serum vitamins and covariates

Variates	*OR*(95%*CI*)	*p*-value
Vitamin D	**0.978(0.954, 1.003)**	**0.079**
Vitamin E	1.002(0.942, 1.066)	0.947
Vitamin B1	**1.116(1.013, 1.231)**	**0.026**
Vitamin B3	1.008(0.994, 1.022)	0.271
Vitamin B6	1.001(0.974, 1.029)	0.922
Vitamin B9	**0.968(0.943, 0.993)**	**0.012**
Age	0.946(0.922, 0.970)	0.000
Sex	0.015(0.008, 0.028)	0.000
Smoking status	1.206(0.297, 4.889)	0.794
Drinking status	1.537(0.486, 4.860)	0.465
Leisure-time physical activity	0.489(0.325, 0.736)	0.001
Nutritious supplementary	1.001(0.509, 1.968)	0.998
Chronic diseases	0.538(0.355, 0.815)	0.003

Supplement Table S6. FDR correction for subgroup analyses (ordinal logistic regression)

	Vitamin	Original *p*	FDR-adjusted *p*	Significant after FDR (α=0.05)
**＜30 years**	Vitamin D	0.335	0.467	No
	Vitamin E	0.908	0.908	No
	Vitamin B1	0.039	0.135	No
	Vitamin B3	0.264	0.467	No
	Vitamin B6	0.045	0.135	No
	Vitamin B9	0.389	0.467	No
**30~55 years**	Vitamin D	**0.001**	**0.006**	**Yes**
	Vitamin E	0.748	0.748	No
	Vitamin B1	0.080	0.160	No
	Vitamin B3	0.352	0.434	No
	Vitamin B6	0.362	0.434	No
	Vitamin B9	0.021	0.063	No
**Male**	Vitamin D	**0.000**	**0.000**	**Yes**
	Vitamin E	0.724	0.724	No
	Vitamin B1	0.361	0.724	No
	Vitamin B3	0.668	0.724	No
	Vitamin B6	0.701	0.724	No
	Vitamin B9	0.363	0.724	No
**Female**	Vitamin D	0.101	0.202	No
	Vitamin E	0.246	0.295	No
	Vitamin B1	0.098	0.202	No
	Vitamin B3	0.164	0.246	No
	Vitamin B6	0.875	0.875	No
	**Vitamin B9**	**0.005**	**0.030**	**Yes**

Supplement Table S7. Stepwise changes in the coefficient of vitamin B9 in linear regression models predicting ASM/BMI

Model	Adjustment	B(95%CI)	*p*-value
1	Unadjusted	-0.001(-0.003, 0.001)	0.391
2	+Age	0.000(-0.002, 0.002)	0.832

3	+Age +Gender	**0.002(0.001, 0.003)**	**0.003**
4	+Age +Gender +Lifestyle factors	0.002(0.001, 0.003)	0.003
5	+Age +Gender +Lifestyle factors+ Nutritional supplement	0.002(0.001, 0.003)	0.005
6	+Age +Gender +Lifestyle factors+ Nutritional supplement +Chronic disease	0.002(0.000, 0.003)	0.008

Supplementary Table S8. Interaction between vitamin B9 and sex in the fully adjusted linear regression model

Term	B(95%CI)	Standard Error	t	*p*-value	VIF
Vitamin B9	0.002(-0.000, 0.003)	0.001	2.589	0.010	1.346
Gender	0.297(0.259, 0.335)	0.020	15.214	0.000	3.304
Vitamin B9 × Gender	-0.001(-0.004, 0.003)	0.002	-0.372	0.710	3.146

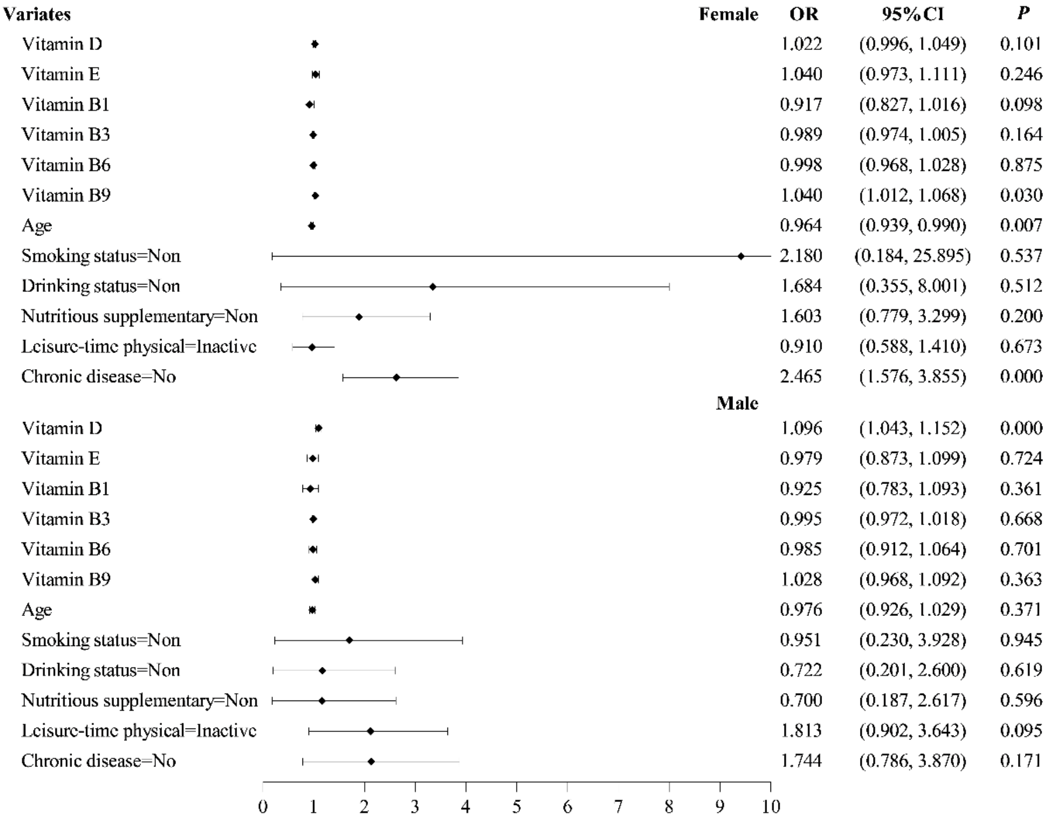

Supplementary Figure S1 Complete forest plots of gender-stratified ordinal logistic regression (all variables).

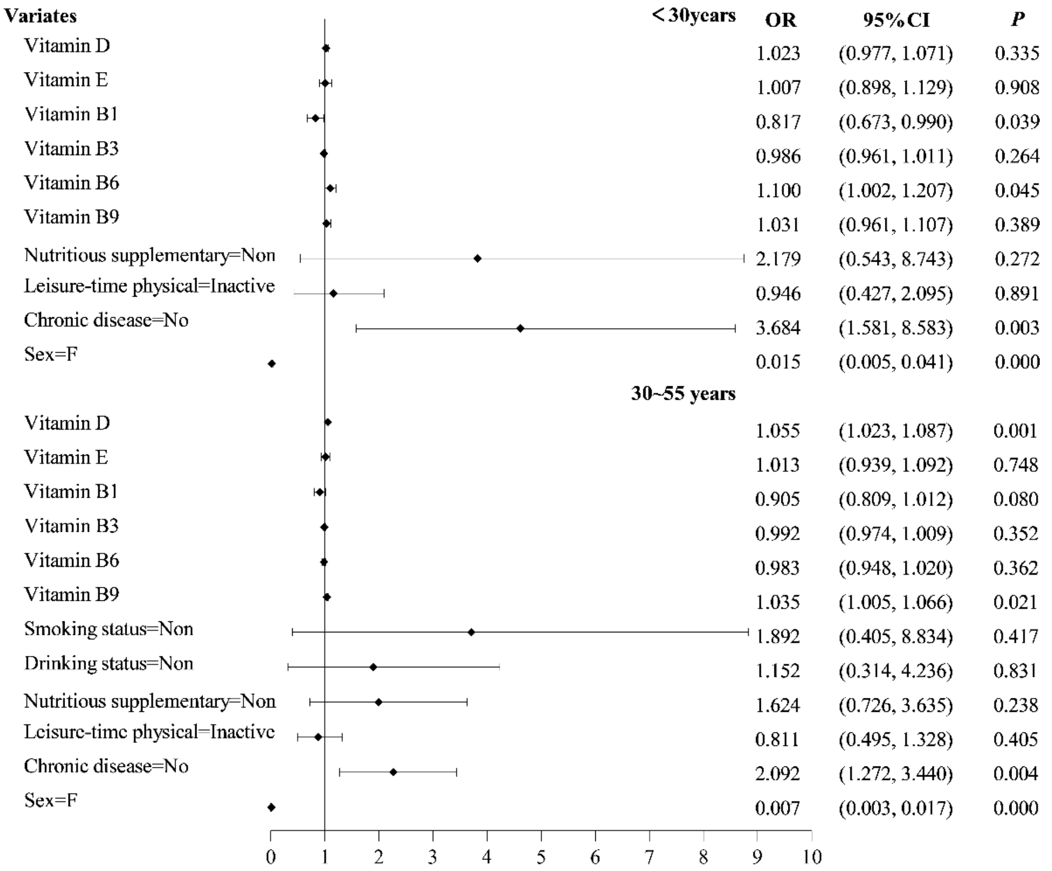

Supplementary Figure S2 Complete forest plots of age-stratified ordinal logistic regression (all variables).
